# Isolation and characterization of plant synergistic bacteria capable of degrading xenobiotics from oil spillage sites

**DOI:** 10.1007/s13205-016-0509-4

**Published:** 2016-09-06

**Authors:** Indranil De, Sarika Gupta

**Affiliations:** 1 Department of Biotechnology, Dr. B. Lal Institute of Biotechnology, Malviya Nagar, Jaipur, 302017 Rajasthan India; 2 Department of Bioscience and Biotechnology, Banasthali University, Banasthali, Tonk, 304022 Rajasthan India

**Keywords:** Xenobiotics, Mung, *Vigna radiata*, Oil spillage, Hydroponics

## Abstract

Oil spillage sites primarily contain various types of hydrocarbons, such as linear chain, polycyclic, and aromatic compounds, posing several detrimental effects on plants. Results from our previous study showed an alteration of various metabolomic parameters, indirectly resulting in an observable decline of growth in the mung seedlings upon incubation with phenol, toluene, xylene, and hexane. This study evaluates the role of these compounds upon plant growth and focusses to mitigate the effect of the same, using some isolated plant synergistic bacteria. We isolated *Proteus* sp., *Streptococcus* sp., and *Enterococcus* sp., and tested the synergism of them in mung seedlings (*Vigna radiata*) by hydroponics. Treatment with the above-mentioned compounds significantly reduced the root and shoot length of the seedlings when compared to the control. The bacterial treatment helped in reducing the adversity due to the xenobiotic insult, by improving the root shoot length of the treated seedlings. *Proteus* sp. was found to be the most promising among other isolates. In another experiment, plasmid profiling of the bacterial isolates was done, yielding a band of 4.5 kb common for all, serving as a clue to be the most probable plasmid responsible for the degradation of the compounds. Results from this study clearly indicate that *Proteus* sp. can be explored further for its plant synergism and xenobiotic degradative capability to exploit its potential in oil spillage land reclamation and establishing vegetation.

## Introduction

The sustainability of population in any part of the world is primarily due to the vegetation it possesses. There are various factors that determine the type of agriculture being done, soil profile being the most important one. Over the years, shifting toward the use of chemical pesticides not only affected the food chain but also upon the soil quality, such as reduced nutrients, water retention capacity. However, certain types of man-made disasters such as petroleum oil spillage create havoc and disrupt the entire agricultural belt along that area. Oil spillage land or petroleum polluted soil mainly contains various polycyclic, linear, and aromatic compounds that hamper the growth of the plants (Wyszkowski and Ziólkowska 2013). In certain cases, it renders the land of no use. Degradation of the hydrocarbons and nutrient cycling can establish vegetation again. In the line of these attempts, people are trying to exploit the potential of soil microbes possessing degradation capabilities of hydrocarbons (Peng et al. 2015). Phenol, toluene, xylene, and hexane are a few hydrocarbons usually present in the spillage sites. Recently, we showed that incubating mung seedlings with these compounds proved to be highly toxic in the case of phenol, followed by hexane, xylene and toluene. Various metabolomic parameters, such as total protein content, total soluble sugar, total reducing sugar, and total phenolic contents, significantly reduced at increasing concentration of the compounds (Gupta and De 2015). In this study, treated mung seedlings with phenol, toluene, xylene, and hexane showed significant reduction in root shoot length as compared to the control group. To counter this effect, isolated bacterial culture was inoculated in the medium, for the degradation of the compound and rendering the seedlings to grow, showing an enhancement of the root shoot length as compared with the treated groups. *Proteus* sp. was found to be the most promising among them. To understand the degradative potential of the bacterial isolates, plasmid profiling was done, yielding a band of nearly 4.5-kb common for all the isolates, serving as a clue to be the most probable plasmid involved in degradation of those compounds. The results from this study clearly indicate that *Proteus* sp. can be explored further in the future for its degradative capability and plant synergism.

## Materials and methods

### Materials

The study was conducted in Dr. B. Lal Institute of Biotechnology, Jaipur. Soil sample was collected from a petroleum station contaminated with petrol and diesel. The mung seeds, *Vigna radiata* Virat HY45, were collected from a certified dealer. All the reagents and solvents were procured from a local supplier, of highest purity.

### Enrichment, isolation, and identification of bacteria

The bacterial suspension was prepared by dissolving 1 g of soil sample in 10 ml of 0.1 % saline solution and thoroughly mixed. The primary enrichment of bacteria was carried out using two sets of trypticase soy broth, one containing sodium azide to permit the growth of Gram positive bacteria and the other containing crystal violet, phenol red to permit the growth of Gram negative bacteria selectively. Each flask was supplemented with 100 ppm of the compound to get only those bacterial isolates capable of degrading them. The flasks were incubated at 37 °C for 24 h in an orbital shaker. The secondary enrichment of bacteria was carried out by streaking the bacterial culture obtained from primary enrichment, on trypticase soy agar plates supplemented with 100 ppm of the compound and incubated at 37 °C for 24 h. The colonies obtained were examined for their morphology and Gram staining was performed to categorize them into Gram positive and Gram negative bacteria. The genus of the isolates was determined using Bergey’s Manual of Systematic Bacteriology through various biochemical characterization test.

### Plasmid profiling of the bacterial isolates

The plasmids of any bacterium host a number of genes responsible for degradation of several hydrocarbons. To gain an insight into the most probable plasmid involved in degradation, they were isolated by alkaline lysis method (Sambrook et al. 1989). The plasmid samples were run on 5 % native gel stained with ethidium bromide. The stained gel was examined under UV light to look for the presence of plasmid bands of particular size using a molecular weight marker (1–6 Kb).

### Assessment of the effect of bacterial isolates on the root shoot length of mung seedlings supplemented with the compound

The degradative potential and plant synergism of the bacterial isolates was determined through hydroponics. The culture bottles were filled with 20 ml of sterilized MS medium supplemented with 100 ppm of compound and each of the bacterial isolates. The mouth of the bottle was covered with aluminum foil with a hole, and the seedling was placed on it touching the medium through its root. They were incubated for 7 days at 26 ± 2 °C, 2000 lux illumination on tissue culture rack. There were two control groups, one containing seedling and medium, another containing seedling, medium, and compound. The test group was inoculated with each of the bacterial isolates in addition to seedling, medium, and compound. The physical health of the seedlings was periodically monitored, and the root shoot length was recorded.

### Statistical analysis

All the experiments were carried out in duplicates and repeated at least two times. The results are presented as mean ± SEM, *n* = 2 from an independent experiment. A two-tailed Student’s *t* test was used for the comparisons between the means of two groups, and *P* values <0.05 were considered as statistically significant.

## Results and discussion

### Bacterial isolates obtained from the soil sample

Bacteria isolated from the petroleum contaminated soil sample is taken, either it is responsible for degradation of hydrocarbons or can utilize it as carbon/energy source or exhibit resistance. The bacteria isolated from the sample were grown in medium supplemented with 100 ppm of compound. We got a total of 20 isolates. On the basis of Gram staining and biochemical characterization followed by validation with Bergey’s Manual of Systematic Bacteriology, the isolates identified were *Proteus* sp. (in hexane), *Streptococcus* sp. (toluene and xylene) and, *Enterococcus* sp. (xylene and phenol).

### Plasmid profiling of the isolates

**Fig. 1:**
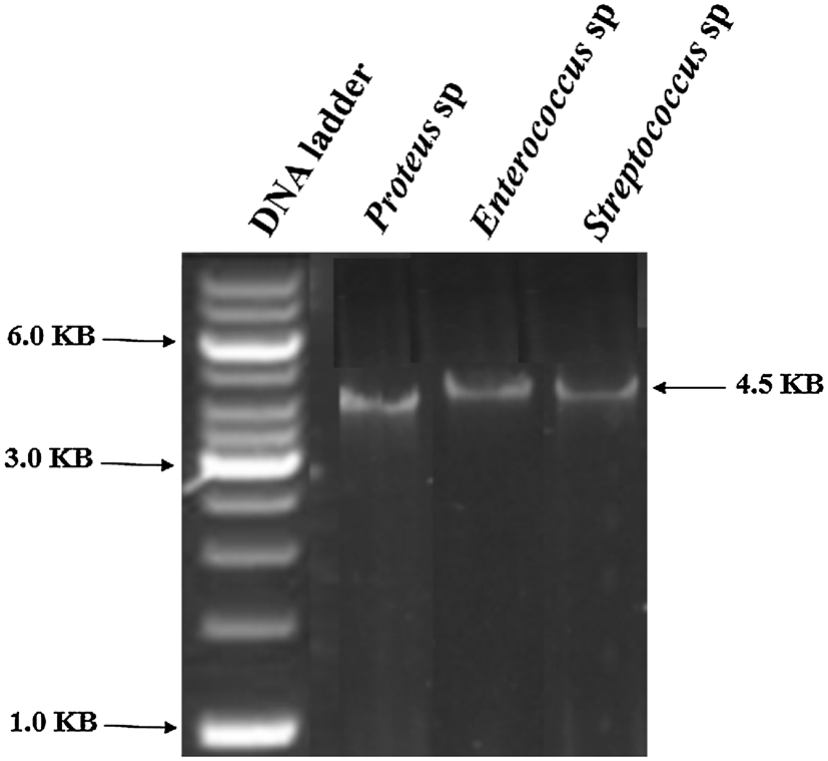
Plasmid of the bacterial isolates. They were isolated by alkaline lysis method followed by running on 5 % native gel and staining it with EtBr. A common band of 4.5 kb of plasmid can be seen in case of all the isolates, hinting to be the plasmid involved in degradation of hydrocarbons

The isolates obtained from the soil sample were proceeded for the plasmid profiling to determine the most probable plasmid involved in the degradation of the hydrocarbons or that is rendering the bacteria to survive under 100 ppm of hydrocarbon stress. A common band of plasmid can be seen common for all the isolates, as shown in Fig. 1.

### Effect of the bacterial isolates on plant growth profile

**Fig. 2:**
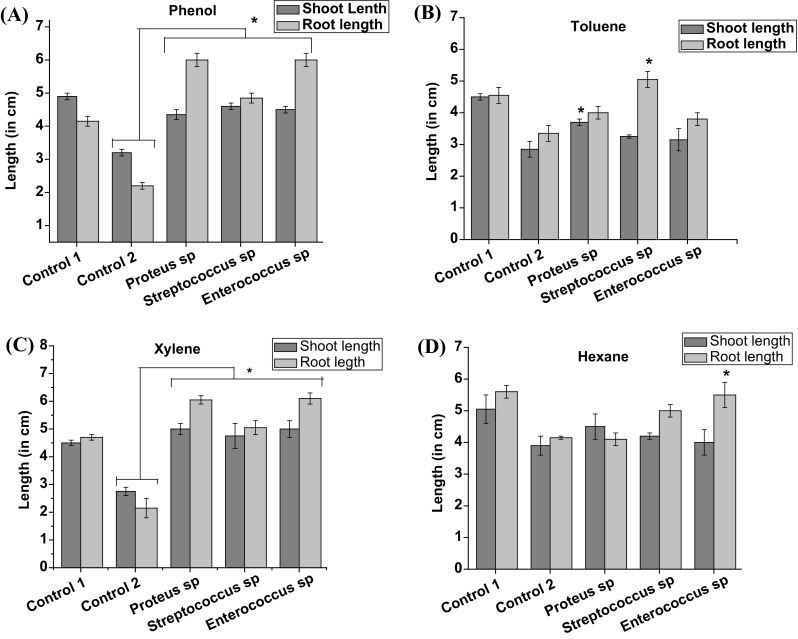
Effect of different bacterial isolates on the root shoot length of the seedlings. Control 1 contains seedling and medium and control 2 contains medium and seedling supplemented with the test compound. The remaining group contains the bacterial isolates inoculated to observe the effect of the isolate in improvement of the root shoot length of the seedling in comparison to control 2. The *graph* shows the effect of bacterial isolates on the seedlings supplemented with **a** phenol, **b** toluene, **c** xylene, and **d** hexane. The results are presented as mean ± SEM (*n* = 2), *P < 0.05 as compared to control 2

To assess the synergism of the bacterial isolates with the seedlings, they were inoculated in the medium and incubated for 7 days with periodic checking. There was a significant decline in the root shoot length of control 2 seedlings in comparison to control 1, as shown in Fig. 2 and Table 1. The bacterial isolates were able to degrade phenol and xylene effectively (Fig. 2a, c) as compared to the toluene and hexane. *Proteus* sp. was found to be able to degrade all the hydrocarbons and establish a better synergism with the seedlings among other isolates.

**Table 1:** Experimental data of the root shoot length of the seedlings in the case of control and the bacterial treated group

Xenobiotic	Shoot root length of seedlings (in cm)	Experimental group				
		Control group		Bacterial treatment group		
		Control 1 (Plant + medium)	Control 2 (Plant + medium + xenobiotic)	*Proteus* sp.	*Enterococcus* sp.	*Streptococcus* sp.
Phenol	Shoot length	4.9 ± 0.1	3.2 ± 0.1	4.35 ± 0.15*	4.5 ± 0.1*	4.6 ± 0.1*
	Root length	4.15 ± 0.15	2.2 ± 0.1	6.0 ± 0.2*	4.85 ± 0.15*	6.0 ± 0.2*
Toluene	Shoot length	4.5 ± 0.1	2.85 ± 0.25	3.7 ± 0.1*	3.25 ± 0.05	3.15 ± 0.35
	Root length	4.55 ± 0.25	3.35 ± 0.25	4.0 ± 0.2	5.05 ± 0.25*	3.8 ± 0.2
Xylene	Shoot length	4.5 ± 0.2	2.75 ± 0.05	5.0 ± 0.2*	4.75 ± 0.45*	5.0 ± 0.3*
	Root length	4.7 ± 0.1	2.15 ± 0.35	6.05 ± 0.15*	5.05 ± 0.25*	6.1 ± 0.2*
Hexane	Shoot length	5.05 ± 0.45	3.9 ± 0.3	4.5 ± 0.4	4.2 ± 0.1	4.0 ± 0.4
	Root length	5.6 ± 0.2	4.15 ± 0.05	4.1 ± 0.2	5.0 ± 0.2*	5.5 ± 0.4*

The results are presented as mean ± SEM (*n* = 2)

* *P* < 0.05 as compared to control 2

The increase in the root length in the presence of *Streptococcus* sp. supplemented with toluene was significant (*P* = 0.02) compared to control 2 (Fig. 2b). In the presence of *Enterococcus* sp., the improvement in the root length of the seedling treated with hexane is also significant (*P* = 0.03) when compared to control 2.

The results and experimental data presented above, clearly reflects *Proteus* sp. to be the most promising one, for plant synergism and hydrocarbon degradation at the same time.

## Discussion

Any form of stress to plants is usually reflected by its alteration in the growth profile, changes in gene expression and metabolomic parameters (Gupta and De 2015). Oil spillage or fuel oil contamination in any area increases the toxic hydrocarbon loads on the soil, which in turn affects the agriculture and vegetation. The hydrocarbons and xenobiotics takes years to degrade, so the process is usually speeded up by bioremediation processes such as using bacteria that can degrade those toxic compounds. For example, *Proteus* sp. can grow on hexane, toluene and able to degrade benzene, toluene, ethylbenzene, and xylene (BTEX) from heavily sites. *Streptococcus* sp. can also degrade phenol from oil contaminated soil (Mohite et al. 2010). *Enterococcus* sp. can grow on hexane, xylene, and degrade azodyes released through textile industry effluent (Mate and Pathade 2012). However, to use the bacterial isolates, we need to test the synergism of them with the plants, so both can coexist together. Results from this study confirm that the bacterial isolates were very well synergistic with the seedlings, and *Proteus* sp. was the best among them. The hydrocarbons degradative potential of any bacteria is mainly attributed to its plasmids present which carries genes for the degradation, for example, *Pseudomonas* sp. possess XYL plasmid that can degrade xylene (Friello et al. 1976). A common molecular weight of 4.5 kb plasmid suggests that it can be the most probable plasmid involved in the degradation of the compounds. To confirm the synergism of bacteria with plant, there must be some enhancement in the growth profile of the stressed plant compared to the normal one. The enhancement in the root shoot length of the seedlings in presence of *Proteus* sp. is statistically significant compared to the control, which suggest it to be an excellent candidate for use, to deal with oil spillage or fuel contaminated soil and restore the vegetation.

## Conclusion

The bacterial isolates were significantly synergistic to the seedlings and possess degradative potential. *Proteus* sp. is the best among others, in terms of the enhancement in root shoot length of the seedlings and degrading the hydrocarbons. *Proteus* sp. can be explored further in the future for its use in fuel contaminated land reclamation.

## References

[CR1] Friello DA, Mylroie JR, Gibson DT, Rogers JE, Chakrabarty AM (1976). XYL, a nonconjugative xylene-degradative plasmid in Pseudomonas Pxy. J Bacteriol.

[CR6] Gupta S, De I​ (2015). Analysis of abiotic stress induced metabolomic changes in vigna radiata. Univers J Environ Res Technol.

[CR2] Mate MS, Pathade G (2012). Biodegradation of CI Reactive Red 195 by *Enterococcus faecalis* strain YZ66. World J Microbiol Biotechnol.

[CR3] Mohite BV, Jalgaonwala RE, Pawar S, Morankar A (2010). Isolation and characterization of phenol degrading bacteria from oil contaminated soil. Innov Romanian Food Biotechnol.

[CR4] Peng M, Zi X, Wang Q (2015). Bacterial community diversity of oil-contaminated soils assessed by high throughput sequencing of 16S rRNA genes. Int J Environ Res Public Health.

[CR5] Sambrook J, Fritsch EF, danManiatis T (1989). Molecular cloning: a laboratory manual.

[CR7] Wyszkowski M, Ziółkowska A (2013). Content of polycyclic aromatic hydrocarbons in soils polluted with petrol and diesel oil after remediation with plants and various substances. Plant Soil Environ.

